# The effects of l-carnitine supplementation on inflammatory factors, oxidative stress, and clinical outcomes in patients with sepsis admitted to the intensive care unit (ICU): study protocol for a double blind, randomized, placebo-controlled clinical trial

**DOI:** 10.1186/s13063-022-06077-3

**Published:** 2022-02-22

**Authors:** Mahdi Keshani, Babak Alikiaii, Gholamreza Askari, Farveh Yahyapoor, Gordon A. Ferns, Mohammad Bagherniya

**Affiliations:** 1 Food Security Research Center, Isfahan University of Medical Sciences, Isfahan, Iran; 2 Department of Community Nutrition, School of Nutrition and Food Science, Isfahan University of Medical Sciences, Isfahan, Iran; 3 Anesthesia and Critical Care Research Center, Isfahan University of Medical Sciences, Isfahan, Iran; 4 Department of Nutrition, Faculty of Medicine, Mashhad University of Medical Sciences, Mashhad, Iran; 5 Brighton & Sussex Medical School, Division of Medical Education, Falmer, Brighton, Sussex, BN1 9PH UK

**Keywords:** l-carnitine, Sepsis, ICU, Inflammation, Oxidative stress, Supplementation, Critically ill

## Abstract

**Background:**

Sepsis is a common cause for admission to the intensive care unit (ICU), and its incidence has been increasing. It is associated with a significant increase in serum inflammatory biomarkers such as C-reactive protein (CRP) and cytokines such as interleukin 1 (IL-1), IL-6, and tumor necrosis factor (TNF). Sepsis is also associated with pathophysiological changes that include fluid accumulation in the lungs, eventually leading to acute respiratory distress syndrome (ARDS), tissue edema, hypotension, and acute kidney injury (AKI). Conventional therapies include antibiotics, but these may have important adverse effects, so novel therapeutic approaches are required. In animal studies, l-carnitine improves antioxidant status, and in some clinical trials, it has been shown to reduce inflammation. It has also been shown to improve respiratory distress and help maintain coenzyme A homeostasis, metabolic flexibility, promoting the normal function of the tricarboxylic acid (TCA) cycle, and oxidation of fatty acids by peroxisomes. We aim to determine the effects of very high doses of l-carnitine on inflammatory factors, oxidative stress, and clinical outcomes of patients with sepsis in ICU.

**Method and design:**

In this double-blind, randomized controlled clinical trial, we will use block randomization of 60 patients with sepsis, aged between 20 and 65 years from Al-Zahra Hospital, Isfahan, Iran. The intervention group (*n* = 30) will receive three capsules of l-carnitine (each capsule contains 1000 mg l-carnitine; totally 3000 mg/day) for 7 days, and a control group (*n* = 30) will receive a placebo with the same dose and for the same duration in addition to usual care. At baseline, scores for clinical and nutritional status (Acute Physiology and Chronic Health Evaluation II (APACHE II), Sequential Organ Failure Assessment (SOFA), Quick SOFA (qSOFA), and NUTRIC Score) will be assessed. At beginning and end point of the study, inflammatory markers (CRP, erythrocyte sedimentation rate (ESR)), oxidative stress status (total oxidative stress (TOS), total antioxidant capacity (TAC)), and clinical variables will be evaluated also. The mortality rate will be assessed within 28 days of the beginning of the intervention.

**Discussion:**

Because of the anti-inflammatory and antioxidant properties of l-carnitine, it is possible that using a high dose of 3000 mg daily of this nutritional supplement may reduce inflammation and oxidative stress and improve subsequent mortality of critically ill patients with sepsis.

**Trial registration:**

Iranian Registry of Clinical Trials IRCT20201129049534N1. Registered on 2 May 2021.

## Administrative information

Note: the numbers in curly brackets in this protocol refer to the SPIRIT checklist item numbers. The order of the items has been modified to group similar items (see https://www.equator-network.org/reporting-guidelines/spirit-2013-statement-defining-standard-protocol-items-for-clinical-trials/).

**Table:** 

Title {1}	The effects of l-carnitine supplementation on inflammatory factors, oxidative stress and clinical outcomes in patients with sepsis admitted to the Intensive Care Unit (ICU): Study protocol for a double blind, randomized, placebo-controlled clinical trial
Trial registration {2a}	IRCT20201129049534N1; 2 May 2021.
Protocol version {3}	Version 1.0; June 2021
Funding {4}	Isfahan university of medical sciences; Grant number: 3991055
Author details {5a}	Mahdi Keshani^1,2^, Babak Alikiaii^3^, Gholamreza Askari^1,2,3^, Farveh Yahyapoor^4^, Gordon A Ferns^5^, Mohammad Bagherniya^1,2,3^
	1. Food Security Research Center, Isfahan University of Medical Sciences, Isfahan, Iran 2. Department of Community Nutrition, School of Nutrition and Food Science, Isfahan University of Medical Sciences, Isfahan, Iran. 3. Anesthesia and Critical Care Research Center, Isfahan University of Medical Sciences, Isfahan, Iran. 4. Department of Nutrition, Faculty of Medicine, Mashhad University of Medical Sciences, Mashhad, Iran. 5. Brighton & Sussex Medical School, Division of Medical Education, Falmer, Brighton, Sussex BN1 9PH, United Kingdom.
Name and contact information for the trial sponsor {5b}	Isfahan university of medical sciences Postal Code: 81746-73461 Tell: (+ 98)-31-3668-0048
Role of sponsor {5c}	Financial supports and supervision

## Introduction

### Background and rationale {6a}

Sepsis is often associated with an immunological reaction. It can result in organ malfunction and eventually death [1]. The immune system recognizes pattern recognition receptors of infective pathogens (PARPs), and the stimulation of the immune system leads to the secretion of pro-inflammatory cytokines such as interleukin 1 (IL-1) and tumor necrosis factor-α (TNF-α) and anti-inflammatory biomarkers. The normal response to infection results in a balance between these inflammatory molecules. In severe sepsis, this equilibrium is put out of kilter and may cause “life-threatening organ dysfunction” [2].

The incidence of sepsis has recently increased globally and is the leading cause of death on, and admission to, intensive care units (ICUs) [3]. More than 30 million people are affected by sepsis worldwide yearly, and this leads to 6 million deaths. The incidence of septic shock and severe sepsis in the USA is approximately 300/100,000 per year and cost more than $20 million in 2011 [4]. The mortality rates in severe sepsis were almost 41% in Europe against around 28.3% in the USA (The Surviving Sepsis Campaign 2012) [1].

Several adverse outcomes are attributed to the sepsis, including endothelial damage (arising from abnormal homeostasis and leading to fluid leak), edema of tissue, hypotension, vascular vasodilatation, and diminished tissue perfusion. Fluid accumulation in the lung can adversely affect gas exchange and lead to acute respiratory distress syndrome (ARDS). Acute kidney injury (AKI) can result from direct effects of cytokines, injury to the microvasculature or tissue perfusion reduction, and a dysregulated coagulation system [2]. Pro-inflammatory cytokines and inflammatory biomarkers, such as C-reactive protein (CRP), IL-1, IL-6 and TNF-α, increase after the development of sepsis [5–7]. Most common therapeutics strategies for controlling this condition are antimicrobials therapies such as antibiotics, antifungals, and antivirals, fluid therapy, vasopressor therapy, ventilation support, activated protein C, and other adjunctive therapies such as corticosteroids, vitamin C, and vitamin B1 [8, 9].

The principal problem is a lack of a specific pharmacological therapy for pathophysiology of sepsis, while many investigations carried out over the last 50 years [10]. Thus, there is a need for the development of new treatments, or new adjunctive treatments. The major metabolic consequences of sepsis include hyperglycemia, ketosis, increased plasma free fatty acids (FFAs), and hyperlactatemia. Specifically, hyperlactatemia indicates tissue hypoperfusion and anaerobic metabolism [10].

l-carnitine is a safe and well tolerated food supplement [11]. l-carnitine enhances FFAs transport into the mitochondria, removing their pernicious effects from the cytoplasm, and separating acetate in mitochondria. Thus, it may alleviate some metabolic malfunction in septic patients [10]. In addition, l-carnitine enhanced superoxide dismutase 2 (SOD2) expression and was anti-inflammation and reduced oxidative stress when studied in animal studies. It reduced several inflammatory mediators like nuclear factor kappa B (NF-ĸB), IL-1, and IL-6, and reduced inflammation of organs. In previous clinical trials which were conducted on hemodialysis patients, on septic shock patients, on coronary artery disease patients, and on perioperative atrial fibrillation patients, it has been shown that this nutrient significantly decreased CRP and the mortality rate [11]. l-carnitine at doses > 2 g per day is more effective in reducing inflammation and in improving health status. Reducing oxidation of lipids, enhancing anti-oxidative stress defense system, and metal ion chelating lead to combat oxidative stress by this nutrient [12]. In a previous trial, l-carnitine supplementation among patients admitted to the neonatal intensive care unit (NICU) with respiratory distress syndrome (RDS) resulted in increasing serum carnitine level. In comparison to control group, the ventilator use period and need for surfactant therapy was also reduced [13]. Some other anti-inflammatory agents that may also be used in sepsis include ulinastatin, which is a serine protease inhibitor that can reduce IL-6, IL-8, TNF-α, and CRP and can alleviate injury of mitochondria and has anti-oxidant effects; thymosin α-1 (Tα1), which is a thymic peptide regulates immune system and so can decrease mortality rate in septic patients [14]; and anisodamine, another medicinal treatment choice for septic shock. It improves blood circulation and reduces TNFα in these patients [15]; besides, anisodamine can combat oxidative agents, act as anti-oxidant, and prevent mitochondrial damage [16].

### Objectives {7}

We aim to investigate the effects of high dose l-carnitine supplementation on inflammatory mediators, oxidative stress, clinical outcomes, and the rate of mortality in ICU septic patients. If this trial confirms our hypothesis, supplementation with l-carnitine may be used to improve the effects of existing therapies.

### Study design {8}

A randomized, parallel, two-arm, double-blind and placebo-controlled superiority clinical trial will be implemented.

## Methods: participants, interventions, and outcomes

### Setting {9}

Our study will be performed in Al-Zahra hospital, an academic hospital, affiliated to Isfahan University of Medical Sciences, Isfahan, Iran.

### Eligibility criteria {10}

- Inclusion criteria:
- Septic patients who will be hospitalized in the ICU. Sepsis will be diagnosed using new definitions for sepsis and septic shock (Sepsis-3) by Surviving Sepsis Campaign International Guidelines for Management of Sepsis and Septic Shock: 2016 [17]. Septic patients will be recruited < 24 h after diagnosis
- Age between 20 and 65 years
- Legal guardian writes informed consent
- Exclusion criteria:
- Patients who stay in hospital for < 3 days
- Patients who receive parenteral nutritional support
- Patients who receive enteral or oral nutrition at first but then transfer to parenteral nutrition due to the contraindications
- Patients who will not be able to receive enteral nutrition or those who will not be able to receive enteral nutrition in the future because of incomplete resuscitation and hemodynamic instability or gastrointestinal disorders including nausea, persistent vomiting, ileus, intestinal obstruction, uncontrolled diarrhea (> 500 ml/day), high-output fistula (> 500 ml/day), and intestinal inaccessibility
- Patients with cancer undergoing chemotherapy and use cisplatin and other drugs that have interaction with l-carnitine; phenobarbital and phenytoin, pivalic acid, valproic acid and ifosfamide, and levetiracetam
- Patients undergoing dialysis, severe and progressive septic shock or sepsis, infection processes, DIC (diffuse intravascular coagulation), and any inflammatory interactions that interfere with the intervention process will be excluded
- Pregnancy
- Patients with BMI < 18.5 kg/m^2^
- Patients who require frequent blood transfusions
- Any unwanted side effects in patients after taking a supplement or placebo

### Who will take informed consent? {26a}

At baseline, the principal investigator (MK) will explain the purpose of the trial to the legal guardian and will obtain their written informed consent upon enrollment.

### Additional consent provisions for collection and use of participant data and biological specimens {26b}

In the written informed consent, we include additional item for getting permission for future research opportunities which participants can say no and only taking part to main study.

### Interventions

#### Explanation for the choice of comparators {6b}

Patients in the intervention and control groups will be given the standard treatments. l-carnitine and placebo (maltodextrin) will be used as adjunctive therapy. Intervention and control pill boxes will be produced by the Karen Pharma and Food Supplement Company, Iran, with similar shape, odor, taste, and package and tagged A and B. Maltodextrin was selected as placebo control because this carbohydrate is digested easily and has a similar taste and appearance as l-carnitine, and it does not interfere with the microbial ecology of the gastrointestinal tract or with gut metabolism and function.

#### Intervention description {11a}

In both groups, after hemodynamic resuscitation and stabilization, nutritional support will begin in the first 24–48 h. Nutritional support with 25 kcal/kg of energy will be determined and will be administered as a bolus method, 7 times in 24 h. Patients will receive all common medications and routine treatment and will be visited daily by a physician who will assess gastrointestinal function.

The intervention will be as capsules in the form of 1000 mg of l-carnitine that will be manufactured by Karen Pharma and Food Supplement company, Iran. Placebo capsules of identical color, odor, and taste will also be produced by above mentioned company.

We consider 3000 mg as the appropriate intervention dose because this dose has low adverse effects and studies show that supplementation > 2 gr/day might have a greater effect [18–20]. l-carnitine and placebo will be tagged A and B by Karen Pharma and Food supplement company and prescribed to the patients using a double-blind procedure. Placebo or l-carnitine will be administered orally or with enteral nutrition (enteral tube feeding) three times a day in 9:00, 15:00, and 21:00 o'clock for 7 days (patients will receive totally 3000 mg l-carnitine or placebo for 7 days).

#### Criteria for discontinuing or modifying allocated interventions {11b}

Though a limited adverse impact has been reported [21], if there are any adverse reactions to the intervention, the intervention will be discontinued and reported to the Isfahan University of Medical Sciences Ethics Committee for a decision. If any participants or their legal guardians ask to cease interventions, we will finish those.

#### Strategies to improve adherence to interventions {11c}

MK will implement the trial and prescribe drugs orally or via enteral tube feeding and another investigator (BA) ask him to return the pill box daily and will check the number of capsules into the pill box. At the end of the study, if the patients received less than 80% of capsules, he/she will be excluded from the study.

#### Relevant concomitant care permitted or prohibited during the trial {11d}

Supplementation with placebo or l-carnitine will not require alteration to usual care pathways (including use of any medications). ‍On the other hand, supplementation with placebo or l-carnitine will not require alteration to usual care pathways (including use of any medications).

#### Treatment strategies

- Controlling source: After sample culture drainage, the clinician prescribes antibiotics (broad-spectrum), antiviral, and or antifungal agents especially in immunodeficiency patient. Then, antimicrobial agents will be limited according to the result of the culture for controlling the source of the infection [9]
- Fluid therapy: For ameliorating hypotension and decreased mean arterial pressure (MAP), the Surviving Sepsis Campaign (SSC) recommended that 30 mg/kg fluid should be administered initially [2]
- Target blood pressure: Low MAP is related to more mortality rate and kidney injury; therefore, for MAP < 65 mmHg vasopressor therapy should be initiated [8]
- Other supportive agents: Many auxiliary agents have been researched such as nutritional antioxidants (vitamin C and vitamin B_1_ [9], glutamine, arginine, and selenium [17]), corticosteroids, and immunoglobulins [2]

#### Provisions for post-trial care {30}

Although no risk of harm will consider for our intervention, the patients will be followed for 1 week after intervention.

#### Outcomes {12}

All assessors in this trial will be blinded to intervention allocation.

Serum CRP and ESR as an inflammatory agent, serum lactate dehydrogenase (LDH) as a marker of cell injury, total oxidative stress (TOS), and total antioxidant capacity (TAC) as an oxidative stress biomarker will be measured as our primary outcomes. Secondary outcomes include evaluating duration of hospitalization (days) and 28-day mortality rate, differences in albumin (Alb), alanine aminotransferase (ALT), aspartate aminotransferase (AST), blood urea nitrogen (BUN), and creatinine in blood. Anthropometric variables such as weight, height, body mass index (BMI), mid arm circumference (MAC), and calf circumference will be appraised at baseline.

Estimation of patient height: Due to the limitations in the ICU, the patients’ height is calculated based on the following formula [22]:

$$ \mathrm{Height}\ \left(\mathrm{cm}\right)=153.492-\left(7.97\times \mathrm{sex}\ \left[\mathrm{sex}:\mathrm{man}=1,\mathrm{woman}=2\right]\right)+\left(0.974\times \mathrm{ulna}\ \mathrm{length}\ \left[\mathrm{in}\ \mathrm{cm}\right]\right) $$

Estimation of patient weight: Due to the limitations in the ICU, the patients’ weight is calculated based on Chumlea I formula [23]:

$$ \mathrm{Men}:\left(\mathrm{MAC}\times 2.31\right)+\left(\mathrm{calf}\ \mathrm{circumference}\times 1.50\right)-50.10 $$

$$ \mathrm{Women}:\left(\mathrm{MAC}\times 1.63\right)+\left(\mathrm{calf}\ \mathrm{circumference}\times 1.43\right)-37.46 $$

Blood drawing will be executed at baseline and end of the trial. About 10 ml volume of fasted blood (at 6:00 o’clock before first meal) will be collected and then centrifuged, with the serum separated from the sediment, and preserved at temperature of − 20 °C.

Calf circumference method of measurement: The circumference of the leg muscle is measured so that the patient is in the supine position. The patient’s left knee rises high enough to form a right corner between the thigh and the leg. We place the tape around the leg muscle and move it along the leg muscle to meter the largest circumference without any pressure to the subcutaneous tissue [24].

### Participant timeline {13}

Study Flow Diagram

**Table:** 

Activity	Time (month)											
	1	2	3	4	5	6	7	8	9	10	11	12
Providing materials	*	*	*	*								
Entrance of patients					*	*	*					
Implementation								*	*			
Analysis of data										*		
Reporting final paper											*	*
Total	*	*	*	*	*	*	*	*	*	*	*	*

### Sample size {14}

The number of samples considering the type I error *α* = 0.05 and the type II error *β* = 0.20 with 80% test power and standardized effect size equal to *Δ* = 30 of the following formula based on to detect the inflammation effect based on changes in CRP (as the main outcome of this study) by l-carnitine supplementation (control group: 99.96 ± 33.16, intervention group: 98.48 ± 37.01) using the findings of Noormandi et al. CRP index (mg/dl) [25]:

$$ n=\frac{2\left[{\left({Z}_{1-\frac{\alpha }{2}}+{Z}_{1-\beta}\right)}^2\times {S}^2\right]}{\varDelta^2}=\frac{2\left[{\left(1.96+0.84\right)}^2\times {(37)}^2\right]}{30^2}=23.85 $$

The required sample size in each group has been equal to 24 people, and with considering the drop out patients, the sample size will be practically 30 patients in every group, which we will consider a total of 60. It seems that this sample size is small; however, several articles which conducted among ICU patients used a small sample size based on a formula like our ones and worked on about 60 patients [26–28].

### Recruitment {15}

Patients will be recruited from the specimens admitted to Al-Zahra hospital, Iran, Isfahan, a referral center in central of Iran.

### Assignment of interventions: allocation

#### Sequence generation {16a}

Each patient will be recruited into intervention or control group using a random number generating website: https://www.sealedenvelope.com/simple-randomiser/v1/lists. Eligible patients will be randomly allocated in a ratio of 1:1 to receive l-carnitine or placebo with a block size of four. Allocation sequence will be determined by sequence in permuted blocks of four. A block size of four is constructed 6 different block to assign patients to the trial groups as follows equally:

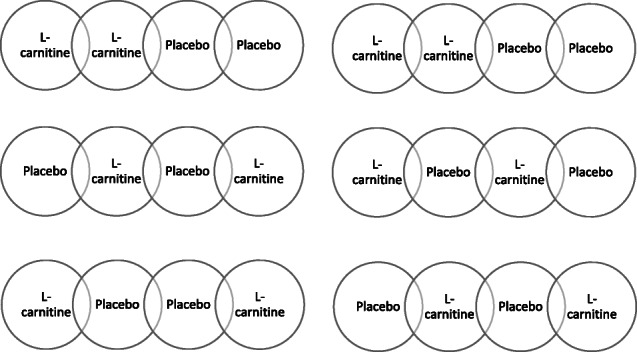



#### Concealment mechanism {16b}

Sealed, opaque envelopes prepared by the supplement manufacturer, along with similar pill boxes that only varied in label (A or B) containing capsules will be used. These are provided to the MK without specifying which package is the l-carnitine or the placebo. The content of envelope determines A or B.

#### Implementation {16c}

Generation of the allocation sequence will be implemented by second investigator (BA) of study and enrollment of participants, and assignment of participants to intervention or control group will be done by the MK.

### Assignment of interventions: blinding

#### Who will be blinded {17a}

l-carnitine and placebo pill boxes will be tagged A and B by the food supplement company (Karen Co.) along with opaque and sealed envelopes, and will be prescribed to participants with a double-blind method. Note that patients or their legal guardians, researchers, and data analysts will be blinded. The data coordinator and trial steering committee will have access to group allocations by accessing to concealment sealed envelopes.

#### Procedure for unbinding if needed {17b}

After implementing statistical analyzes, we will open Karen supplement company envelopes to reveal labels. Any unbinding will be updated on Iranian Registry of Clinical Trials and reported.

### Data collection and management

#### Plans for assessment and collection of outcomes {18a}

Inflammatory biomarkers will be evaluated with ELISA assay method (CRP, ESR, and LDH), oxidative stress will be determined by spectrophotometric methods using commercial kits, and BUN, creatinine, and Alb will be measured at the Clinical Chemistry Laboratory in Al-Zahra hospital, according to a standardized protocol. Anthropometric variables also will be evaluated by MK according to the mentioned formula using a tape meter.

#### Plans to promote participant retention and complete follow-up {18b}

Not applicable.

#### Data management {19}

Data will be collected by the MK on paper sheets and will be entered to Microsoft Excel software by another blinded team researcher and then also will be random-checked by third team member.

#### Plans for collection, laboratory evaluation, and storage of biological specimens for genetic or molecular analysis in this trial/future use {33}

Blood samples will be collected by investigators, will be labeled with subject ID, and then immediately will be sent to the central laboratory (Alzahra Clinical Research Laboratory, Isfahan) for keeping in − 80 °C freezers. After collecting all specimens, laboratory assessment methods will be executed. Storing of biological specimen will be done only if participants accept the secondary informed consent. No genetic studies are currently planned.

### Statistical methods

#### Statistical methods for primary and secondary outcomes {20a}

Analyzing data will be performed in the SPSS 16 (version) software. Normality will be checked by Kolmogorov-Smirnov test. Qualitative data will be reported as frequencies, normal continuous quantitative as mean (± SD) and median (IQR) for others.

Differences within group will be assessed with paired *t*-test and Wilcoxon rank-sum test.

Between group differences also will be assessed using independent *t*-test and Mann-Whitney *U* test. The significance level will be considered *p* < 0.05.

#### Interim analyses {21b}

An independent statistician will carry out an interim analysis (the logistic regression model) for safety and adverse events when we have reached 50% of the events between two groups. Then, the results will be evaluated by the trial steering committee.

#### Methods for additional analyses (e.g., subgroup analyses) {20b}

No subgroup analyses are planned.

#### Methods in analysis to handle protocol non-adherence and any statistical methods to handle missing data {20c}

Intention-to-treat analysis will be carried out for missing data. Patient exclusion reasons will be reported completely. We plan to perform sensitivity analyses to explore the effect of departures from the missing data assumptions made in the efficacy analyses. Multiple imputation will be used for missing data.

#### Plans to give access to the full protocol, participant level-data and statistical code {31c}

After checking rational demands for additional analyses, the corresponding author will make datasets available.

### Oversight and monitoring

#### Composition of the coordinating center and trial steering committee {5d}

The Ethics Committee and Vice Chancellery of Isfahan University of Medical Sciences supervise all the study stages. They will oversee the conduct of the trial at any unexpected time and will issue recommendations for early termination, modifications, or continuation of the trial, if necessary.

#### Composition of the data monitoring committee, its role and reporting structure {21a}

The composition of committee includes committee director, committee secretary, and other science committee whom will oversee the implementation of study continuously. It is an academic committee that sponsors this trial and has no competing interest.

#### Adverse event reporting and harms {22}

Although many studies indicated this normal dosage of l-carnitine has no considerable adverse effects, it might have some minor side effects such as diarrhea and fish odor syndrome [21]. Nevertheless, any adverse events that occur during the study will be reported to the Department of Community Nutrition and ethical committee of Isfahan University of Medical Sciences for decision-making.

#### Frequency and plans for auditing trial conduct {23}

The Ethics Committee will be responsible for monitoring the trial. Audits on accuracy may be carried out at any time and at least twice.

#### Plans for communicating important protocol amendments to relevant parties (e.g., trial participants, ethical committees) {25}

Every change in the study will be reviewed by the trial steering committee and updated in https://irct.ir/ and will be informed to Trials Journal also.

#### Confidentiality {27}

Each participant in trial will be received a unique ID, and the patient identification information will not be exposed to anyone except the trial team. Written informed consent, data papers, and computer system that will be used for storing data will be locked by the first author.

#### Dissemination plans {31a}

The final study results will be published in official publications.

## Discussion

l-carnitine (lysine + methionine) is involved in beta-oxidation [29]. Its anti-inflammatory and antioxidant effects have already proven though its effects on septic patients are not conclusive [12, 30]. In a previous study in patients with renal disease, malondialdehyde (MDA) decrease and reduced/oxidized glutathione and glutathione peroxidase activity increase statistically meaningful with l-carnitine supplementation [31].. Hadis Fathizadeh, et al in their meta-analysis demonstrated that l-carnitine supplementation in healthy persons or patients with special disorders can reduce serum inflammatory cytokines such as CRP, IL-6, TNF-α, and MDA, and enhancing SOD levels [30]. Two recent meta-analyses from limited trials demonstrate that l-carnitine can help to reduce the mortality rate in septic patient, but more studies are needed with more rigorous design and different doses and durations [32, 33].

Sepsis is known to impair pro-inflammatory immune response and increase some inflammatory and oxidative biomarkers in blood that spread throughout the world and is a major concern in ICU which has a significant mortality. Because of the aging population, outbreak of some infectious diseases such as COVID-19, and weakening the immune system resulting from inaccurate lifestyle, recent research proved that the sepsis incidence is expanding [34]. In addition, investigations indicate that mitochondrial dysfunction in sepsis is a serious factor in organ failure.

Energy production in mitochondria requires l-carnitine-mediated transport (carnitine palmitoyl transferase 1). Its function is restrained in sepsis [35]. Mitochondrial dysfunction in sepsis is a negative agent in organ failure and is related to sepsis fatality rate. On the other hand, mitochondrial dysfunction signs include acylcarnitine enhancement, malfunction in β-oxidation of fats, and the presence of mitochondrial DNA in blood.

**Fig. 1:**
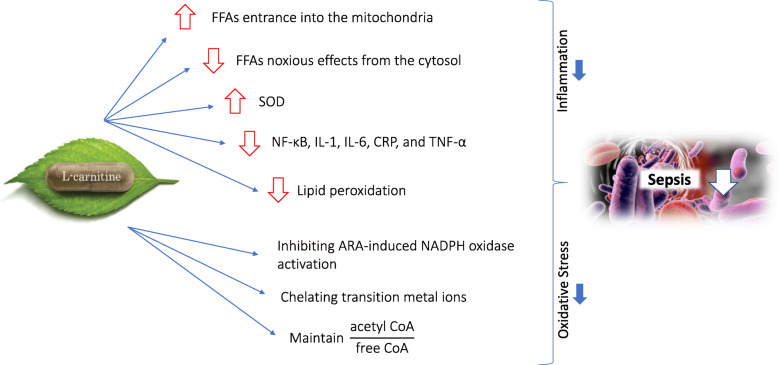
L-Carnitine thorough several pathways might have beneficial effects on the sepsis. It suppresses inflammation, reduces oxidative stress, and modulates metabolism

Antioxidant benefit of l-carnitine is the deactivation of arachidonic acid-induced NADPH oxidase. Evidences in patients with peripheral arterial disease show that l-carnitine was ameliorated arterial dysfunction by decreasing oxidative stress. Additionally, it may regulate oxidative stress and platelet activation in surgical process [36].

l-carnitine maintains acetylated CoA to free CoA ratio within the mitochondria. Failure to hold this ratio can lead to impaired energy production and can accumulate acyl CoA compounds [37]. In many studies, l-carnitine suppressed inflammation and reduced CRP, IL-6, and TNF-α [38–41] (Fig. 1).

Since sepsis has not definite treatment, if l-carnitine supplementation will be beneficial in the handling of sepsis, it could be a new adjunctive therapy in future.

## Trial status

Version 1.0; June 2021. Recruitment will be started in September 2021 and approximately will be completed in March 2022.

## Abbreviations

ICU: Intensive care unit
CRP: C-reactive protein
IL-1: Interleukin-1
TNF: Tumor necrosis factor
ARDS: Acute respiratory distress syndrome
AKI: Acute kidney injury
ESR: Erythrocyte sedimentation rate
TOS: Total oxidative stress
TAC: Total antioxidant capacity
APACHE II: Acute Physiology and Chronic Health Evaluation II
SOFA: Sequential Organ Failure Assessment
qSOFA: Quick SOFA
BMI: Body mass index
MAC: Mid-arm circumference
FFAs: Free fatty acids
SOD: Superoxide dismutase
NF-ĸB: Nuclear factor kappa B
NICU: Neonatal intensive care unit
RDS: Respiratory distress syndrome
DIC: Diffuse intravascular coagulation
MDA: Malondialdehyde
